# PARP-1 overexpression contributes to Cadmium-induced death in rat proximal tubular cells via parthanatos and the MAPK signalling pathway

**DOI:** 10.1038/s41598-017-04555-2

**Published:** 2017-06-28

**Authors:** Tongwang Luo, Yan Yuan, Qi Yu, Gang Liu, Mengfei Long, Kanglei Zhang, Jianchun Bian, Jianhong Gu, Hui Zou, Yi Wang, Jiaqiao Zhu, Xuezhong Liu, Zongping Liu

**Affiliations:** 1 College of Veterinary Medicine, Yangzhou University, 12 East Wenhui Road, Yangzhou, 225009 People’s Republic of China; 2 Jiangsu Co-innovation Center for Prevention and Control of Important Animal Infectious Diseases and Zoonoses, Yangzhou, 225009 People’s Republic of China; 3 Jiangsu Key Laboratory of Zoonosis, Yangzhou, China

## Abstract

Parthanatos is a newly discovered form of PARP-1-dependent programmed cell death. It has been reported to play an important role in several cancer or tumour cells; however, few studies have been performed in normal cells. Cadmium is a highly toxic pollutant and is reported to induce autophagy and apoptosis in multiple cell types. Although cadmium toxicity induces cell death, the underlying mechanism is not fully understood. Therefore, in this study we aimed to investigate the mechanism of Cadmium -induced cell damage using rat proximal tubular cell line NRK-52E and primary rat proximal tubular (rPT) cells. Our results indicated that parthanatos and the MAPK signalling pathway contribute to Cadmium-induced cell death, and that oxidative stress and mitochondrial damage play key roles in this process. In addition, parthanatos with oxidative stress has a synergistic effect on apoptosis, and JNK1/2 and p38 contribute to parthanatos.

## Introduction

Cadmium (Cd) is a widespread toxic metal in the environment that originates mainly from industry and agriculture^1^. Cd causes serious harm to humans and livestock when it becomes bio-magnified in food webs. There have been reports of Cd contamination events in recent years worldwide^2, 3^. Our laboratory has long been committed to investigating the mechanism of cadmium toxicity. We and others have found that Cd can not only accumulate in the body and affect the body’s growth and reproduction, but also can lead to severe oxidative stress, cell autophagy, and apoptosis. However, the underlying mechanism of Cd-induced cell death remains poorly understood.

Parthanatos is a recently discovered Poly (ADP-ribose) synthetase 1 (PARP-1)-dependent form of cell death^4, 5^, in which the excessive activation of PARP-1 resulting in poly ADP ribose (PAR) accumulation in the cytoplasm, causing mitochondrial permeability changes. This consumes large amounts of ATP and NAD, leading to disruption of necessary intracellular biochemical reactions^5^, thereby causing cell death. PARP-1 is a multifunctional, post-translationally modified enzyme that is found widely in eukaryotic cells^6, 7^. Under physiological conditions, PARP-1 is important for the repair of DNA damage, genome stability, apoptosis, and gene transcription^8^. However, when excessively activated, PARP-1 plays prominent roles in many diseases, such as stroke, Parkinson’s disease, heart failure and diabetes^9^. Therefore, control of the potential parthanatos target sites could not only inhibit this method of cell death, but also could ameliorate related diseases, which is one of the purposes of this study. The family of mitogen-activated protein kinases (MAPK) and their signalling pathways are involved in cell growth, proliferation, differentiation, and apoptosis^10, 11^. Among them, the ERK MAPK pathway is involved mainly in cell proliferation, at the same time, studies have shown that the high activation of ERK is also involved in the process of cell damage and caused cell apoptosis^12^. JNK MAPK and p38 MAPK pathways can be activated under stress conditions, they are involved in cell apoptosis signal, growth inhibition signal and inflammatory response^13^. ERK1/2 and JNK1/2 MAPK can mediate the downstream signals of PARP-1. Indeed, PARP-1 activation causes the phosphorylation of ERK1/2 and Bax^14^. When PARP-1 activity is disrupted by inhibitors, the amount of activated caspase-3 protein and the number of dead cells are reduced, in addition, JNK1/2 and ERK1/2 protein can be used as the upstream factor of PARP-1 to regulate cell death^15, 16^. Therefore, we speculated that the MAPK pathway is involved in Cd-induced renal injury.

Currently, there are few studies on parthanatos and its mechanism of action is not clear. Thus, we wished to determine whether Cd-induced rat renal tubular epithelial cell damage involves parthanatos and the MAPK apoptosis pathways, and whether there is a connection between them. Therefore, we used NRK-52E cells and primary rPT cells as models to explore whether Cd can induce PARP-1-dependent cell death via parthanatos and to explore the relationship between the parthanatos and MAPK pathways.

## Materials and Methods

### Chemicals and antibodies

All of the chemicals were the highest grade available. SP600125, SB203580, NAcetyl-L-cysteine (NAC) (purity of ≥99%), 3, 4-Dihydro-5-[4-(1-piperidinyl)butoxy]-1(2H)-iso-Quinoline (DPQ), and cadmium acetate (CdAc2) were purchased from Sigma-Aldrich (St. Louis, MO, USA). Dulbecco’s modified Eagle’s medium (DMEM)-F12 (1:1), Opti-MEM I Reduced Serum Medium, fetal bovine serum (FBS), trypsin-EDTA, collagenase IV, and Lipofectamine 3000 Transfection Reagent were obtained from Thermo Fisher Scientific (Waltham, MA USA). DAPI (2-(4-amidinophenyl)-1H-indole-6-carboxamidine) was from Sigma-Aldrich. The Cell Counting Kit-8 (CCK-8) was from Dojindo Laboratories (Tokyo, Japan). The Annexin V-FITC apoptosis detection kit and mitochondrial membrane potential (JC-1) assay kit were purchased from BD Biosciences (San Diego, CA, USA). The NAD^+^/NADH Assay kit was purchased from Suzhou Ered Biological Technology Co. Ltd (Suzhou, China). The ATP Assay Kit and redox-sensitive dye DCFH-DA were obtained from Beyotime Biotechnology Co. Ltd (Shanghai, China). The scrambled short interfering RNA (siRNA) and PARP-1 siRNAs were synthesized by Invitrogen (Shanghai, China).

Rabbit anti-Histone-3H (CST, 9718S), anti-cleaved caspase-3(CST, 9664S), anti-cleaved caspase-9 (CST, 9507), anti-ERK1/2 (CST, 4695S), anti-phosphotyrosine ERK1/2 (CST, 4370S), anti-JNK1/2 (CST, 9252S), anti-phosphotyrosine JNK1/2 (CST, 4668S), anti-p38 (CST, 8690S), anti-phosphotyrosine p38 (CST, 4511S), anti-cytC (CST, 11940S), anti–COX IV (CST,4890S), anti-β-actin (CST, 4970S) and horseradish peroxidase (HRP)-conjugated goat anti-rabbit immunoglobulin G (IgG) antibodies were obtained from Cell Signaling Technology Inc. (Danvers, MA, USA). Anti-AIF antibody (abcam, ab110327), anti-Bax antibody (abcam, ab32503), anti-Bcl-2 antibody (abcam, ab136285) were purchased from Abcam Ltd (Cambridge, MA, USA). Anti-PARP-1 antibody (Santa, sc-7150) was purchased from Santa Cruz Biotechnology (Santa Cruz, CA, USA). Mouse anti-PAR polymer antibody (USBio, 045159) was obtained from Ed Technology Co (Beijing, China). The dilution of the antibodies were according to the instructions.

### Cell culture and Cd treatment

The NRK-52E cell line was purchased from the Cell Bank of the Institute of Biochemistry and Cell Biology (Shanghai, China), and was used for no more than 15 passages. Cells were cultured in DMEM supplemented with 10% FBS and incubated at 37 °C in a 5% CO_2_ atmosphere. The primary rPT cells were obtained from the kidneys of Sprague-Dawley rats with body weights between 180 and 200 g. The Rats were purchased from the Laboratory Animal Center at Yangzhou University (Yangzhou, China). The identification and culture of the cells were performed according to previous descriptions^17, 18^. The purity of the isolated primary rPT cells was >95% and the cells were subcultured using 0.25% trypsin-0.02% EDTA digestion. Cells cultured for 12 h had the highest viability (according to the growth curve, data not shown). Furthermore, based on the doses used in a previous study^19^, the rPT cells were treated with 1.25, 2.5, or 5.0 μM Cd. The Cd acetate stock solution was dissolved in sterile ultrapure water.

All the experimental procedures in this study were approved by the Animal Care and Use Committee of Yangzhou University (Approval ID: SYXK (Su) 2012–0029), and this study was carried out in accordance with the Guide for the Care and Use of Laboratory Animals by the National Research Council.

### Cell proliferation and viability measurement

NRK-52E cell proliferation was monitored using the real-time cell analysis (RTCA) system (Roche, Mannheim, Germany), according to the manufacturer’s instructions. First, we determined the background level by loading 100 μL/well of culture medium (DMEM with 10% FBS) into a 16-well E-plate, and then, 100 μL of the NRK-52E cell suspension (approximately 10,000 cells) was added in each E-plate well. Cells were incubated for 30 min in an incubator and then cell proliferation was measured every 15 min. Cells were treated at the exponential growth stage with serum-free culture medium containing different compounds according to the experimental design; each group contained four wells.

The rPT Cells (2 × 10^4^ cells/well) were seeded onto 96-well plates and grown to almost 90% confluence, after which they were treated with a series of Cd concentrations for 4, 6, 8, and 12 h. At each time point, cell viability assays were performed using the CCK-8 kit according to the manufacturer’s instructions and the absorbance were read at 450 nm using a microplate reader (Sunrise, Austria).

### Western Blotting Analysis

Cells were processed according to the experimental design requirements and the proteins were extracted by ultrasonication in radio-immunoprecipitation assay lysis buffer (Beijing Applygen Technologies Inc) and a protease inhibitor cocktail. Total proteins (20–40 μg) were separated on 6–15% SDS polyacrylamide gels and transferred to 0.22 μm polyvinylidene fluoride or nitrocellulose membranes. The membranes were then incubated with blocking solution containing 5% non-fat milk for 2 h at room temperature (24–26 °C). The membranes were then incubated overnight at 4 °C with the relevant primary antibodies. Thereafter, the membranes were washed and incubated with the appropriate secondary antibodies (1:5000 dilution) for 2 h at room temperature. The membranes were then washed six times with TBST, with shaking for 5 min each time, and the immunoreactive protein bands were detected by enhanced chemiluminescence reagents. Finally, the immunoreactive protein bands were quantified using the Image Lab software (Bio-Rad, Hercules, CA, USA). The proteins volumes were determined by standard scanning densitometry and the density of each band was normalized to that of the β-actin., COX IV, or Histone-3H control. All assays were performed in triplicate.

### Immunofluorescence Assays

Primary rPT cells were seeded at a density of 1 × 10^6^ cells/well in six-well plates and treated with or without 2.5 μM Cd for 12 h. After washing in PBS, the cells were fixed on coverslips with 4% paraformaldehyde, permeabilized with 0.5% TritonX-100 for 15 min, and blocked with 5% BSA for 90 min. The cells were then incubated overnight with primary antibodies in blocking solution at 4 °C, washed with PBS, and stained with the appropriate fluorescent secondary antibodies at room temperature for 1 h. The cells were then co-stained with DAPI to visualize the nuclear morphology. Fluorescence images were captured using a laser scanning confocal microscope (TCS SP8 STED; Wetzlar, Hessen, GER).

### Detection of Cellular ATP Levels

Cellular ATP levels were measured using an ATP assay kit according to the manufacturer’s instructions. After the indicated treatments, the cells were homogenized in an ice-cold ATP-releasing buffer and centrifuged at 12,000 × g for 5 min. The ATP levels in the supernatant were then analysed according to the manufacturer’s instructions. The luminescence from a 50 μL sample mixed with 100 μL of ATP detection working dilution in a white 96-well plate was assayed in a single-tube luminometer (Turner Biosystems, Sunnyvale, CA, USA). Standard curves were generated using known concentrations of ATP, and the ATP concentration of each treatment group was determined using the standard curves. The experiment was repeated three times and total ATP levels were expressed as nmol/mg protein.

### NAD^+^ Determination

We measured the intracellular NAD^+^ concentration using an NAD^+^ assay kit according to the manufacturer’s instructions. The detailed procedure was as follows: cells were seeded into 12-well plates at a concentration of 5 × 10^5^ cells/well with 500 μL culture media per well. After treatment, cells were washed with cold PBS and collected in Eppendorf tubes and the cells were pelleted. Each pellet was resuspended in 100 μL of extraction buffer and heated for 5 min at 60 °C. Then, 20 μL of assay buffer and 100 μL of opposite extraction buffer were added to neutralize the extracts. Cells samples were centrifuged at 20000 × g for 5 min, and the supernatants were collected for NAD^+^ assays. After mixing the working reagent (a mixture of 60 μL assay buffer, 1 μL enzyme A, 1 μL enzyme B, 14 μL lactate, and 14 μL MTT ((3-(4,5-dimethylthiazol-2-yl)-2,5-diphenyltetrazolium bromide)), the absorbance of the standards and samples was measured immediately at 565 nm (OD_0_), as well as 15 min later (OD_15_). We used the ΔOD values (OD_15_–OD_0_) to determine the NAD^+^ concentration in samples from the standard curve.

### Mitochondrial membrane potential (JC-1) assay

The NRK-52E and primary rPT cells were treated with Cd for 12 h at the indicated doses alone or pretreated for 30 min with 25 μM DPQ, followed by Cd incubation. The cells were then collected and stained with JC-1 according to the instructions of the mitochondrial membrane potential (JC-1) assay kit. JC-1 is a dye and in normal mitochondria it is present as a multimer and shows red fluorescence. When the mitochondrial membrane is lost, JC-1 monomers will increase and green fluorescence is enhanced. The percentage of cells that exhibited a decreased mitochondrial membrane potential was determined by gating the population of cells with a decrease in JC-1 aggregates and a simultaneous increase in JC-1 monomers. After the cells were stained with JC-1, the NRK-52E cells and primary rPT cells were analysed by flow cytometry (CyAn ADP 7; Brea, CA, USA).

### Measurement of intracellular ROS

The average level of intracellular ROS in NRK-52E cells and primary rPT cells was evaluated using the redoxsensitive dye DCFH-DA. The level of intracellular ROS was examined at the indicated time points for cells treated with Cd for 12 h alone or combined with DPQ or PARP-1 siRNA. All experimental samples were washed twice in PBS and stained in the dark for 30 min with 20 μmol/L DCFH-DA. The cell samples were then dissolved with 1% Triton-100, and the fluorescence was measured at an excitation wavelength of 485 nm and an emission wavelength 530 nm using flow cytometry (CyAn ADP 7; Brea, CA, USA).

### Apoptosis detection by flow cytometry

Cells were plated in six-well plates and incubated. After being treated according to the experimental design, the apoptotic cells were evaluated using an Annexin-V/FITC Apoptosis Detection Kit according to the manufacturer’s protocol and the cells were analysed by flow cytometry (CyAn ADP 7; Brea, CA, USA).

### Acridine orange-Ethidium bromide (AO-EB) staining assay

In the AO-EB staining assay, AO can penetrate intact cell membranes and becomes embedded in nuclear DNA, where it binds double-stranded DNA and emits green fluorescence. EB can only penetrate damaged cell membranes to embed in nuclear DNA where it emits orange-red fluorescence. Apoptotic cells show enhanced staining, more luminous fluorescence, and a uniform round shrinkage-like or lump-like structure. Non-apoptotic nuclei exhibit a structure-like feature with different shades of fluorescence. Thus, under the fluorescence microscope four cell morphologies are observed. Live cells: the cells nuclear chromatin is green has a normal structure. Early apoptotic cells: the cells nuclear chromatin appears green and show shrinkage-like or fragment-like morphologies. Late apoptotic cells: the cells nuclear chromatin appear orange-red and showed shrinkage-like or fragment-like structures. Non-apoptotic dead cells: the cells nuclear chromatin appears red and shows a normal structure.

To investigate whether N-acetylcysteine (NAC) has a protective effect on Cd-induced apoptosis or necrosis, the nuclear morphology were analysed with AO-EB staining. AO-EB staining was assessed at the indicated time points for cells treated with Cd alone or combined with 100 μM NAC for 12 h. Five microlitres of 100 μg/mL AO-EB was added to the live cells at room temperature in the dark and then observed under a fluorescence microscope (Leica 2500; Leica Corporation, Germany).

### Small interfering RNA (siRNA) transfection

NRK-52E cells were grown in a culture dish with diameter of 10 cm. Transfection of PARP-1 siRNA into the cells was performed by using Lipofectamine 3000, according to the manufacturer’s protocol. The siRNA targeting PARP-1 was 5′-GAGCACUUCAUGAAAUUAUTT-3′. After siRNA transfection for 24 h, the cells were incubated with 5 μM Cd 12 h for subsequent experiments.

### Statistical Analysis

All data represent at least three independent experiments and the mean ± SD value was determined by one-way analysis of variance (ANOVA) using the SPSS 22.0 statistical software (SPSS, Chicago, IL, USA). The results were considered statistically significant at P < 0.05 and highly statistically significant at P < 0.01.

## Results

### Cd inhibited the proliferation and viability of cells

**Figure 1:**
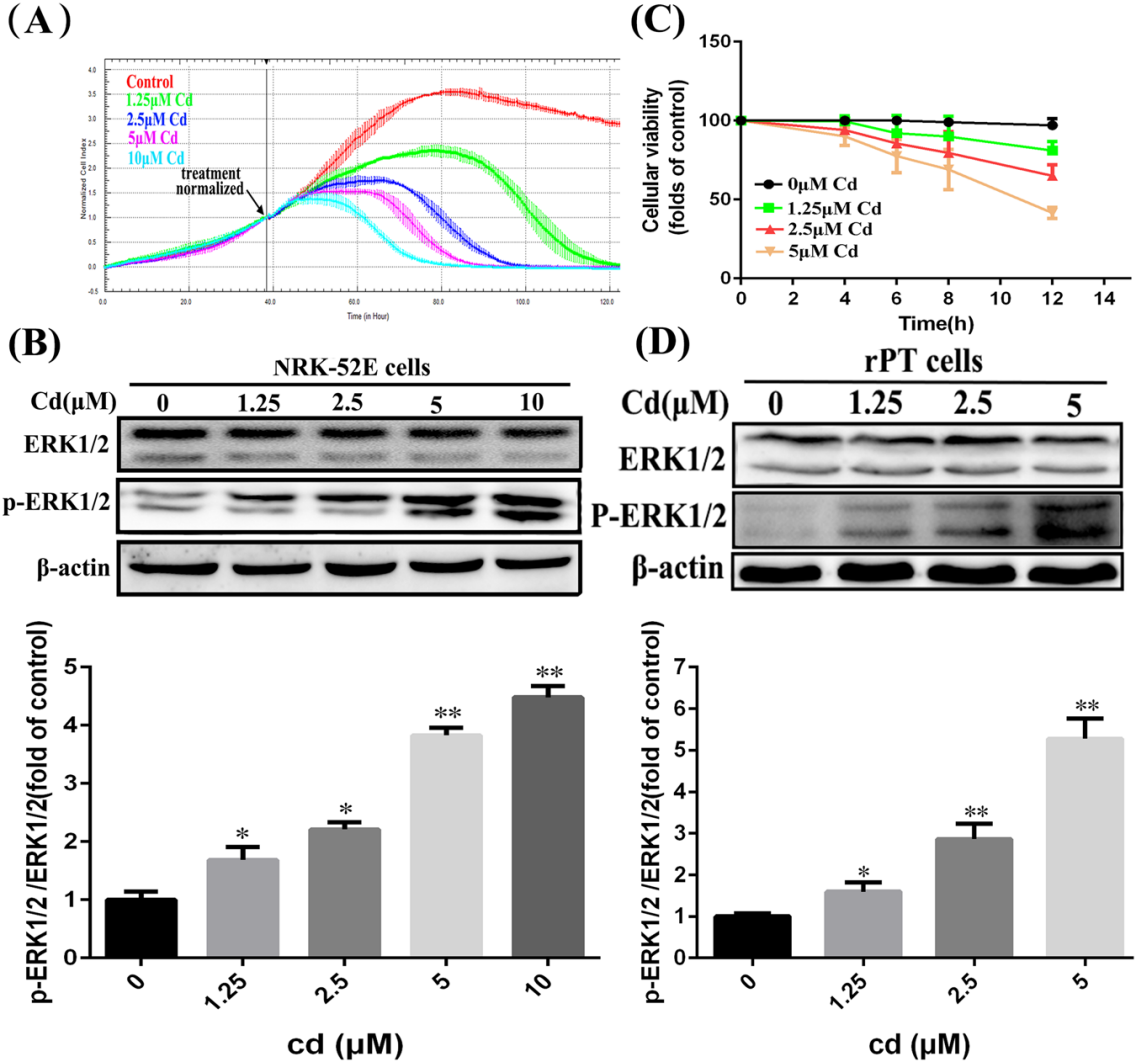
Effects of cadmium on cell proliferation and survival. (**A**) Cd inhibited the proliferation of NRK-52E cells. Normalized cell index were determined by RTCA after treated with 1.25, 2.5, 5 or 10 μM Cd and Error bars indicate SD (n = 3). (**B**) Western blot analysis of the expression of ERK1/2 and p-ERK1/2 protein in NRK-52E cells after treated with 1.25, 2.5, 5 or 10 μM Cd for 12 h, and the rate of p-ERK1/2/ERK1/2 was significantly increased in 5, 10 μM Cd group that compared with the control group. (**C**) Cell counting kit-8 method to detect the effect of Cd (1.25, 2.5, 5 μM) on the proliferation of primary rPT cells, and compared with the control group, the survival rate of primary rPT cells decreased gradually. (**D**) Western blot analysis of the expression of ERK1/2 and p-ERK1/2 protein in primary rPT cells after treated with 1.25, 2.5 or 5 μM Cd for 12 h, and the rate of p-ERK1/2/ERK1/2 was significantly increased in 2.5, 5 μM Cd group that compared with the control group. Semi-quantitative analysis was performed with images from three independent experiments (mean ± SD, n = 3). *P < 0.05; **P < 0.01 vs. the control group by one-way ANOVA.

To investigate the effect of Cd on renal tubular epithelial cell proliferation and its toxic effect, we observed the growth of NRK-52E cells at different Cd concentrations in real-time using RTCA. At the same time, we used CCK-8 to detect the viability of primary rPT cells at various Cd concentrations for different periods. The RTCA results (Fig. 1A,C) indicated that Cd inhibited the proliferation of NRK-52E cells dose-dependently within a certain range. Correspondingly, the CCK-8 results showed that compared with the control group, the survival rate of primary rPT cells decreased gradually as Cd the concentration and the exposure time increased. In addition, we detected the levels of ERK-MAPK-related proteins in cells (Fig. 1B,D), which indicated that the ratio of p-ERK1/2 to ERK1/2 increased significantly with increasing Cd concentration in both NRK-52E cells and primary rPT cells. These results showed that Cd not only inhibited the viability of renal tubular epithelial cell, but also activated the ERK MARK pathways.

### Cd caused the upregulation of PARP-1 and induced parthanatos in rPT cells

**Figure 2:**
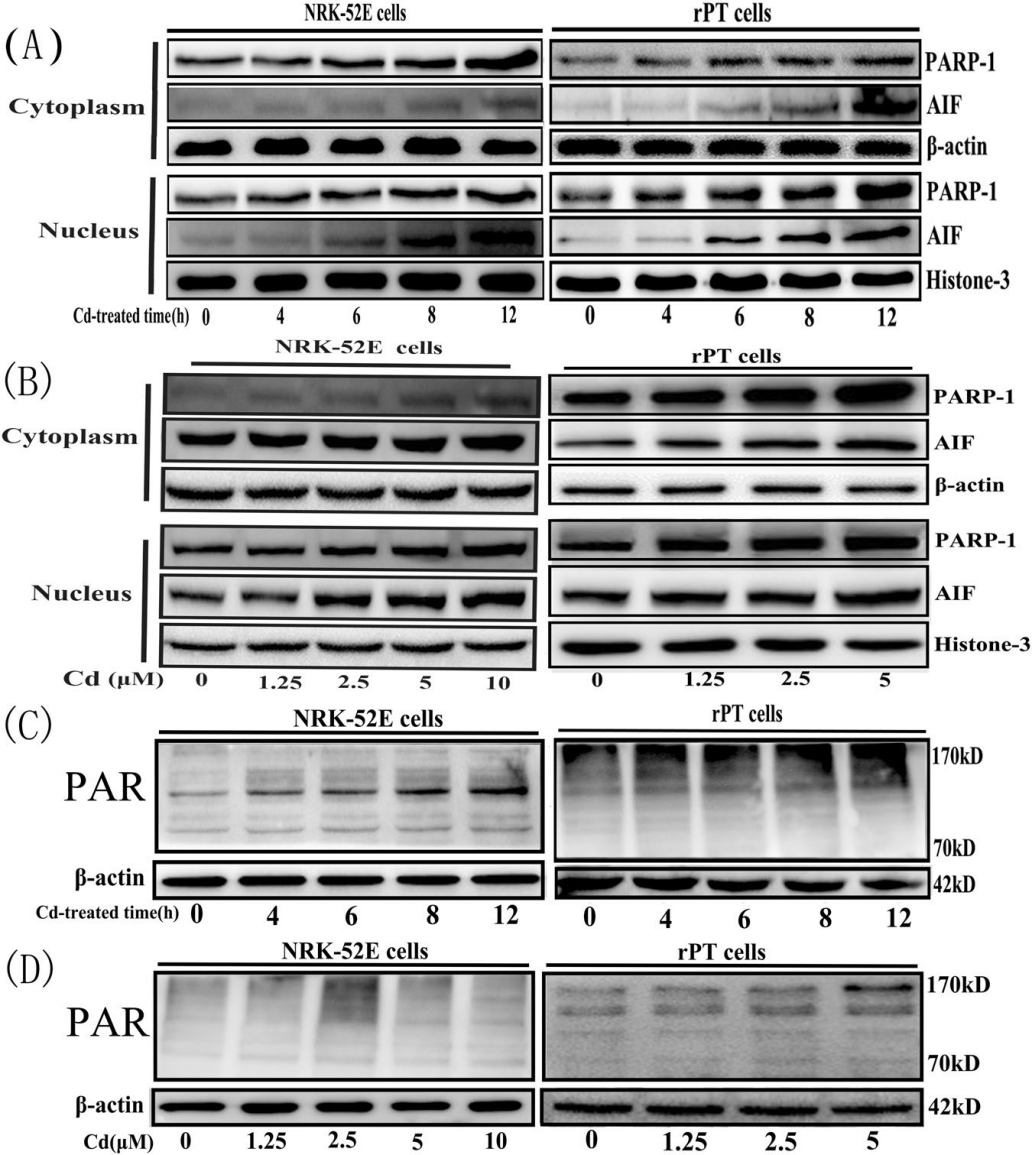
Changes of parthanatos-related proteins caused by cadmium. Western blot analysis of the expression of parthanatos-related proteins in NRK-52E cells and primary rPT cells after treated with different concentrations of Cd for 12 h. (**A** and **B**) Cytoplasmic and nuclear PARP-1 and nuclear AIF increased as the Cd concentration and incubation time increased in NRK-52E cells and primary rPT cells. (**C** and **D**) Cd induced the cytoplasmic formation of PAR polymer, dependent on its concentration and exposure time in NRK-52E cells and primary rPT cells.

**Figure 3:**
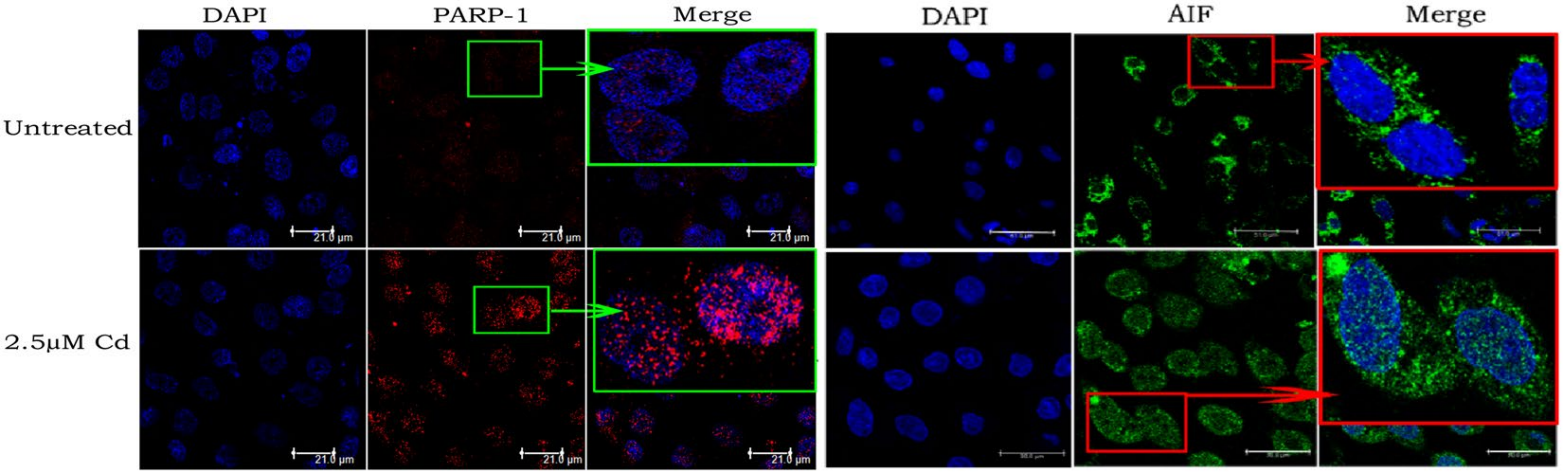
PARP-1 accumulation and the nuclear translocation of AIF in primary rPT cells, as detected by immunofluorescence. Primary rPT cells were treated with or without 2.5 μM Cd for 12 h and were stained to visualize PARP-1 and AIF Protein. Nuclear accumulation of active PARP-1 protein (Red) and AIF (green) translocation into the nucleus were obvious.

**Figure 4:**
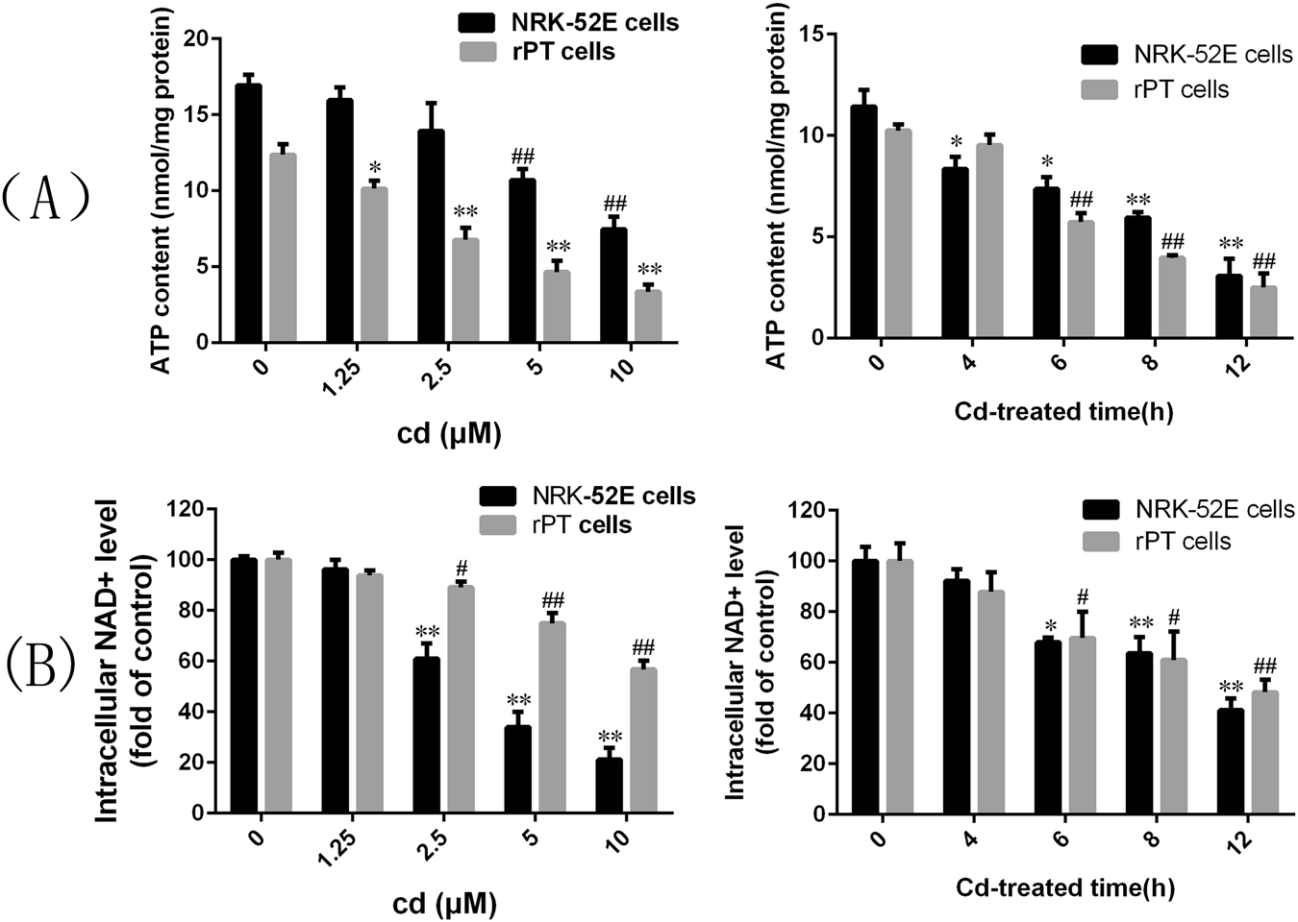
Effects of cadmium on ATP and NAD^+^ contents in NRK-52E and primary rPT cells. (**A**) Effects of cadmium on the ATP content in NRK-52E and primary rPT cells. With the increase of Cd concentration and the prolongation of exposure time the contents of ATP presents decreased trend. (**B**) Effects of cadmium on the NAD^+^ content in NRK-52E and primary rPT cells. With the increase of Cd concentration and the prolongation of exposure time the contents of NAD^+^ presents decreased trend. Data are expressed as mean ± SD (n = 3). *P < 0.05; **P < 0.01; ^#^P < 0.05; ^##^P < 0.01 vs. the control group by one-way ANOVA.

To investigate whether parthanatos contributes to the renal tubular epithelial cell death caused by Cd, the expression of characteristic proteins in the parthanatos pathway were examined by western blotting. Figure 2 shows that the levels of PARP-1 increased obviously with increasing Cd concentration and prolonged exposure time. The formation of the PARP-1 polymer also showed an increasing trend. Furthermore, the level of the nuclear protein AIF was distinctly higher in the group treated with Cd compared with that in the control group, and increased when the Cd dose was enhanced and the incubation time was prolonged. We then used immunofluorescence to detect the nuclear accumulation of PARP-1 and the nuclear translocation of AIF in rPT cells (Fig. 3). The fluorescence intensity of PARP-1 and PAR in the Cd-treated group was significantly higher than that in the control group and increased nuclear translocation of AIF was also observed in response to Cd, which was consistent with the western blotting results. In addition, we examined the changes in ATP and NAD^+^ levels at different exposure times and concentrations of Cd, as shown in Fig. 4, with prolongation exposure to Cd, the ATP and NAD^+^ contents decreased significantly. The changes of ATP and NAD^+^ contents were also measured after 12 hours of treatment at different concentrations of Cd. The results showed that the ATP and NAD^+^ contents decreased with increasing Cd concentration, and when the concentration of Cd reached 5 μM, the ATP or NAD^+^ contents were decreased significantly compared with the control group.

**Figure 5:**
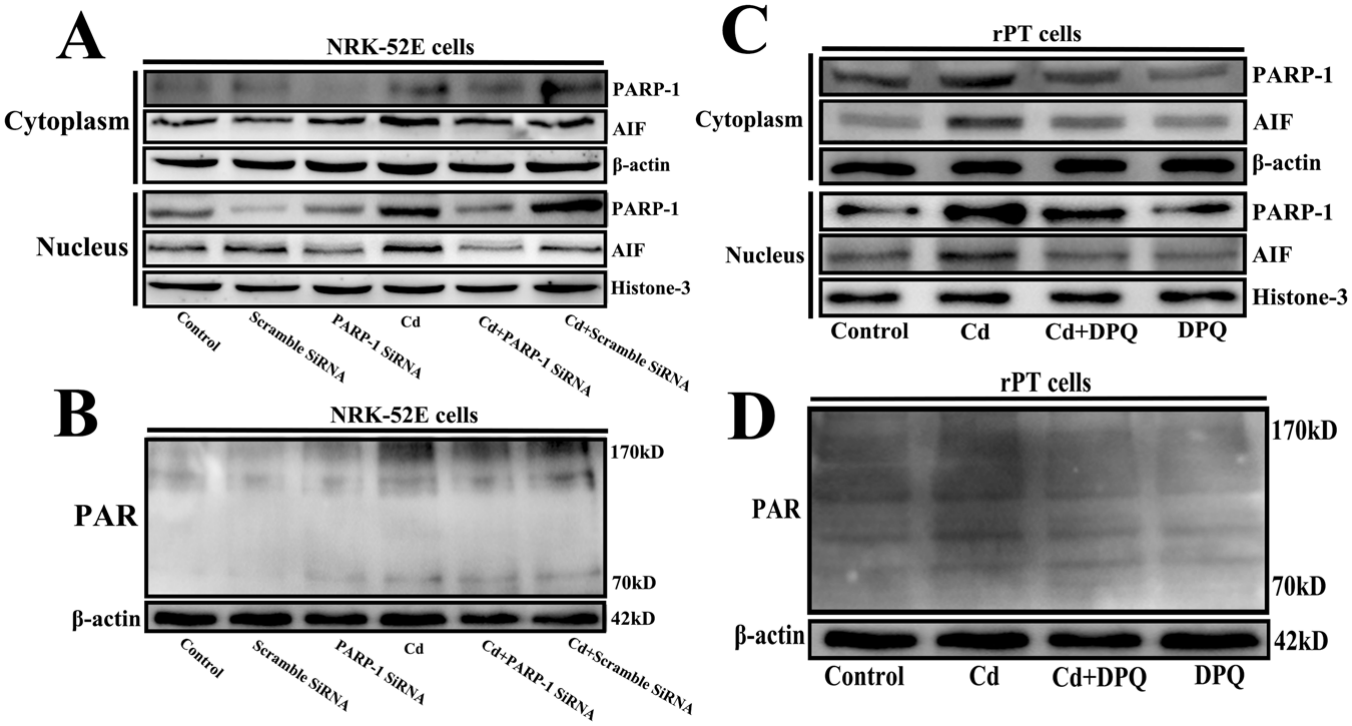
Inhibit the expression of PARP-1 protein attenuated renal tubular epithelial cells death that induced by Cd. (**A** and **B**) Western blotting analysis showed that transfection with PARP-1 SiRNA inhibited the expression of PARP-1, PAR polymer and mitigated the increase of nuclear AIF in NRK-52E cells. (**C** and **D**) Western blotting analysis showed that inhibit the expression of PARP-1 by DPQ reduced the level of PARP-1, PAR polymer and the nuclear AIF in rPT cells.

Besides, in order to further confirm the role of PARP-1 in Cd-induced renal tubular epithelial cells death, we knocked down PARP-1 with siRNA in NRK-52E cells or inhibited the expression of PARP-1 in rPT cells. The results as shown in Fig. 5, when inhibited the expression of PARP-1 in NRK-52E cells or in rPT cells, the level of PARP-1, cytoplasmic PAR polymer and the nuclear AIF were lower, compared with that in the Cd-treated group or scramble siRNA group. This indicated that Cd-induced elevation in both the nuclear level of AIF and the level of cytoplasmic PAR were regulated by PARP-1. These results suggested that Cd treatment can induce PARP-1-dependent parthanatos in renal tubular epithelial cells.

### Cd-induced parthanatos is targeted to the mitochondria and causes apoptosis

**Figure 6:**
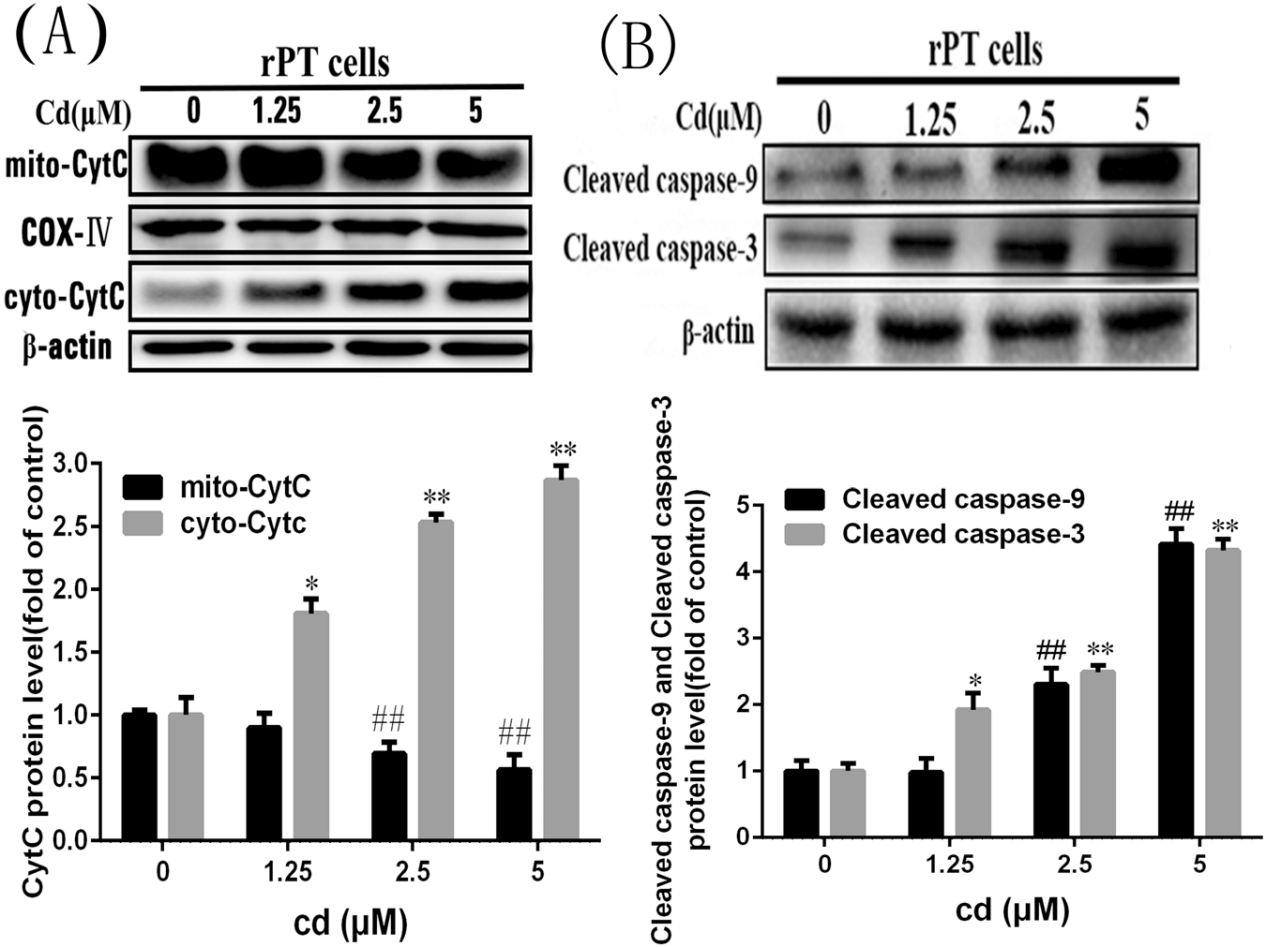
Effect of cadmium on the mitochondrial apoptosis pathway. Western blot analysis of the release of Cyt C protein and the expression of Cleaved caspase-9/3 in primary rPT cells after treated with 1.25, 2.5 or 5 μM Cd for 12 h. (**A**) Compared with the control group, the release amount of Cyt C increased gradually. (**B**) Compared with the control group, the expression of Cleaved caspase-9 and Cleaved caspase-3 increased gradually. The quantitative analysis was performed on the western blotting results of three independent experiments (mean ± SD, n = 3). Respectively, *P < 0.05;**P < 0.01 vs. the control group by one-way ANOVA. ^##^P < 0.01 vs. the Cd group by one-way ANOVA.

**Figure 7:**
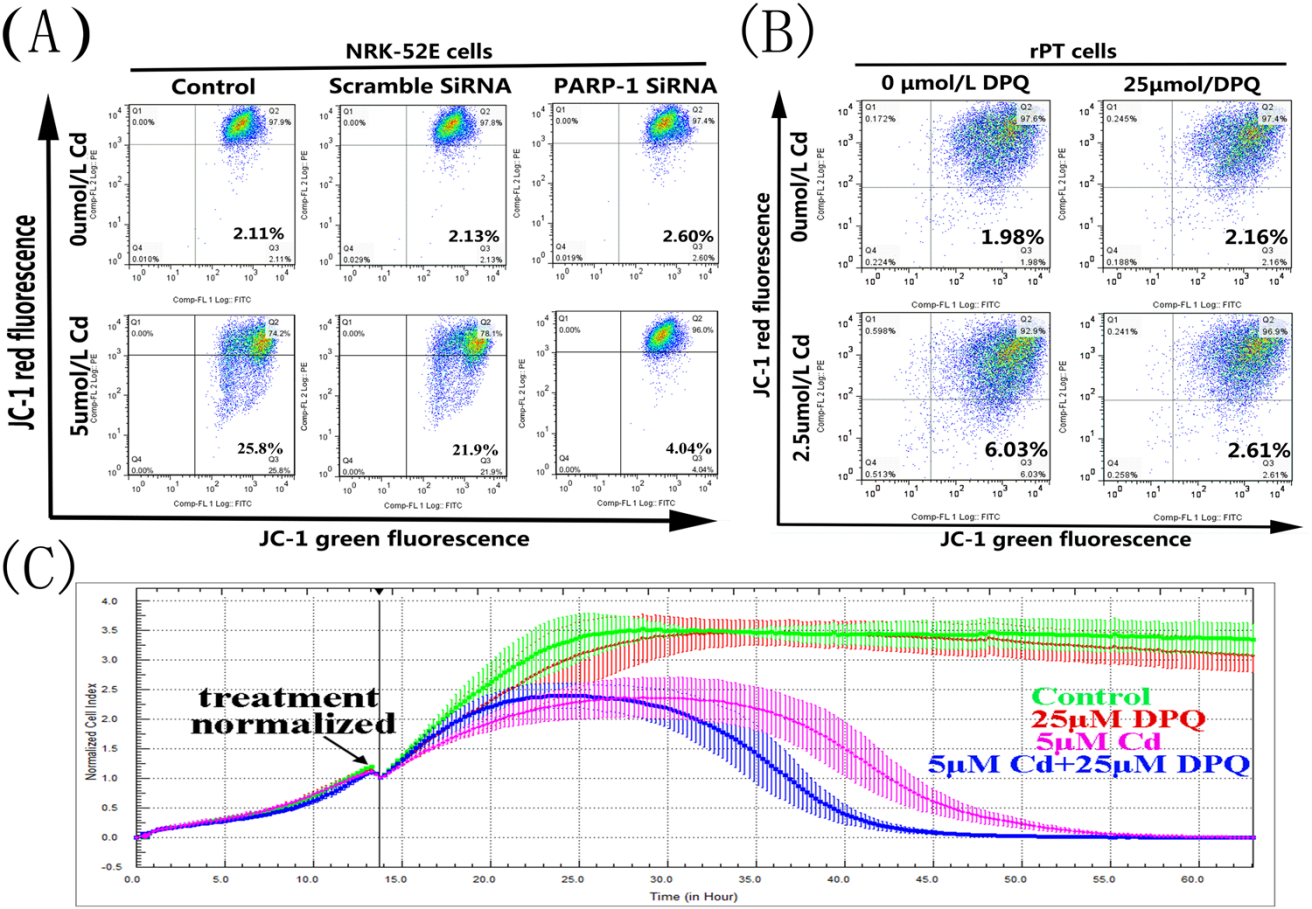
The effect of PARP-1 protein on mitochondrial membrane potential and proliferation of rat proximal tubular cells. (**A** and **B**) Flow cytometry analysis combined with JC-1 staining showed the decline in the mitochondrial membrane potential caused by Cd treatment for 12 hours in rat renal tubular epithelial cells, which was inhibited by PARP-1 gene knockout or PARP-1 inhibitor DPQ at 25 μM. (**C**) Cell index for NRK-52E cells treated with 5 μM Cd and 25 μM DPQ as indicated. Results were normalized to the time of treatment and error bars indicate SD (n = 4).

To investigate whether Parthanatos acts on mitochondria and plays a role in cell damage, we examined the effect of Cd on the mitochondrial apoptosis pathway (Fig. 6). As the Cd concentration increased, the level of CytC in mitochondria decreased obviously and the expression level of CytC in the cytoplasm was increased markedly, and the downstream proteins caspase-9 and caspase-3 were activated. This suggested that Cd activates the mitochondrial apoptotic pathway. We then silenced the *PARP-1* gene or inhibited PARP-1 protein activity and detected changes in the mitochondrial membrane potential. As shown in Fig. 7(A and B), flow cytometry analysis indicated that the red fluorescence (JC-1 multimers) decreased at 12 h both in the NRK-52E cells and primary rPT cells that were incubated with Cd, and the green fluorescence (JC-1 monomers) increased correspondingly. However, when the *PARP-1* gene was silenced in NRK-52E cells or the PARP-1 protein was inhibited in primary rPT cells, the mitochondrial membrane potential in both cells declined compared with the Cd group.

**Figure 8:**
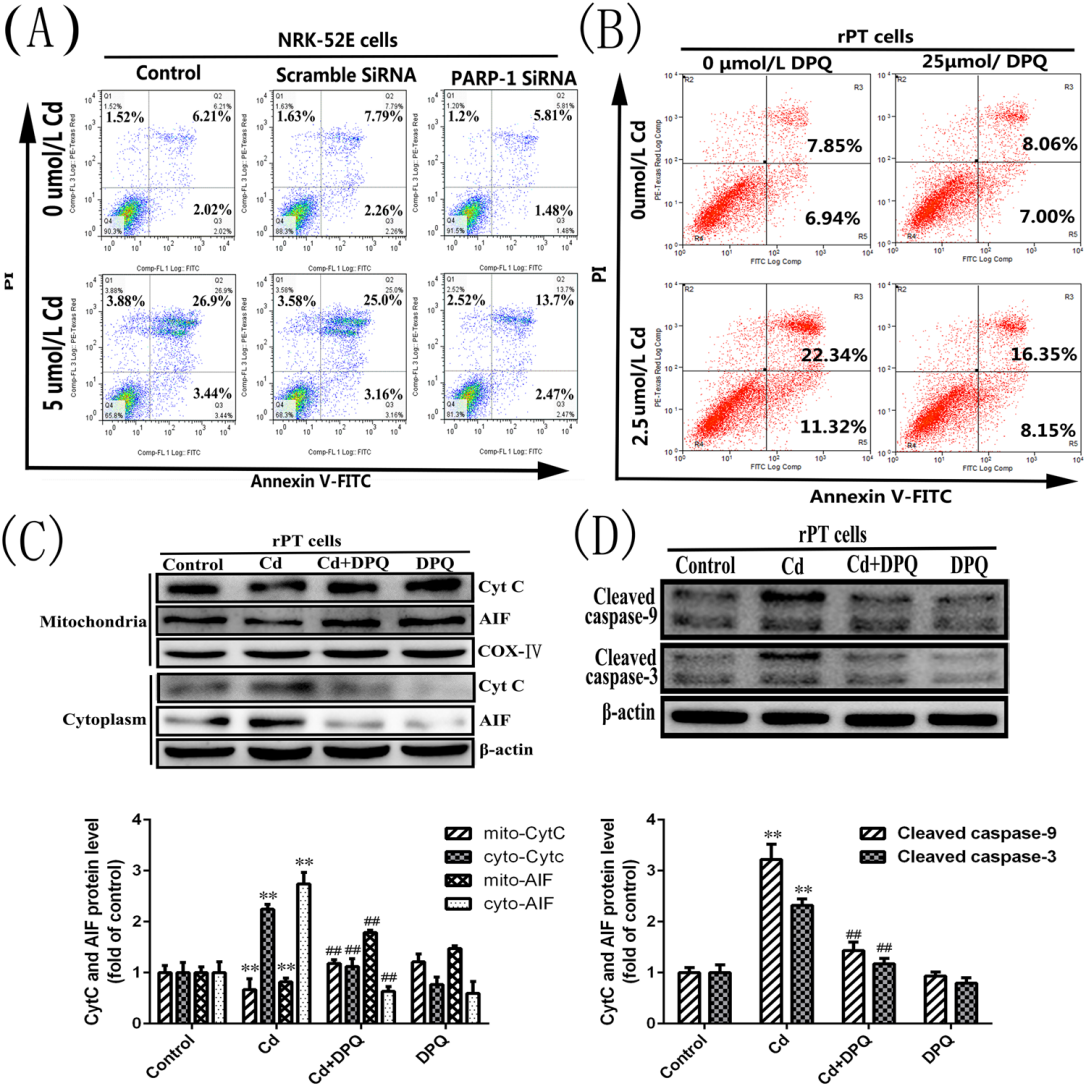
Effects of PARP-1 on Cd-induced apoptosis and expression of related proteins in the mitochondrial apoptosis pathway. (**A** and **B**) After treatment with Cd, the apoptosis rate of NRK-52E cells and primary rPT cells increased, compared with the control group. When the PARP-1 gene was silenced or PARP-1 protein activation was inhibited, the apoptosis rate of Cd-treated NRK-52E cells or primary rPT cells decreased compared with the Cd group. (**C** and **D**) Western blot analysis of the release of Cyt C and AIF and the expression of Cleaved caspase-9/3 in primary rPT cells after treated with Cd and/or DPQ for 12 h. Data are expressed as mean ± SD (n = 3) relative to control. **P < 0.01 in comparison to the control by one-way ANOVA. ^##^P < 0.01 in comparison to the Cd group by one-way ANOVA.

Next, we used RTCA to observe the growth of NRK-52E cells after inhibition of PARP-1 protein activation (Fig. 7C). Cells in which PARP-1 protein over-activation was inhibited by DPQ grew better than those grown in the presence of Cd; however, prolonged Cd exposure proved to be cytotoxic, even in the presence of DPQ. We then investigated the effect of PARP-1 on apoptosis and mitochondria-associated apoptosis proteins. The results indicated that after treatment with Cd, the apoptosis rate of NRK-52E cells increased from 8.23 to 30.34% and the apoptotic rate of primary rPT cells increased from 14.79 to 33.66%. When the PARP-1 gene was silenced or PARP-1 protein activation was inhibited, the apoptosis rate of Cd-treated NRK-52E cells decreased to 16.17% and that of Cd-treated primary rPT cells decreased to 24.5% (Fig. 8A and B). Moreover, the release of CytC and AIF proteins from mitochondria and the levels of cleaved caspase-9 and cleaved caspase-3 all decreased compared with the Cd-alone group (Fig. 8C and D). This suggested that Cd disrupts the mitochondrial membrane potential and leads to cell apoptosis; whereas, inhibition of PARP-1 protein overexpression can mitigate the mitochondrial damage and, to some extent, inhibit the apoptosis induced by Cd. Thus, Cd-induced parthanatos is closely related to mitochondrial apoptotic pathways.

### Cd activates parthanatos via oxidative stress and parthanatos exacerbates oxidative damage to rPT cells

**Figure 9:**
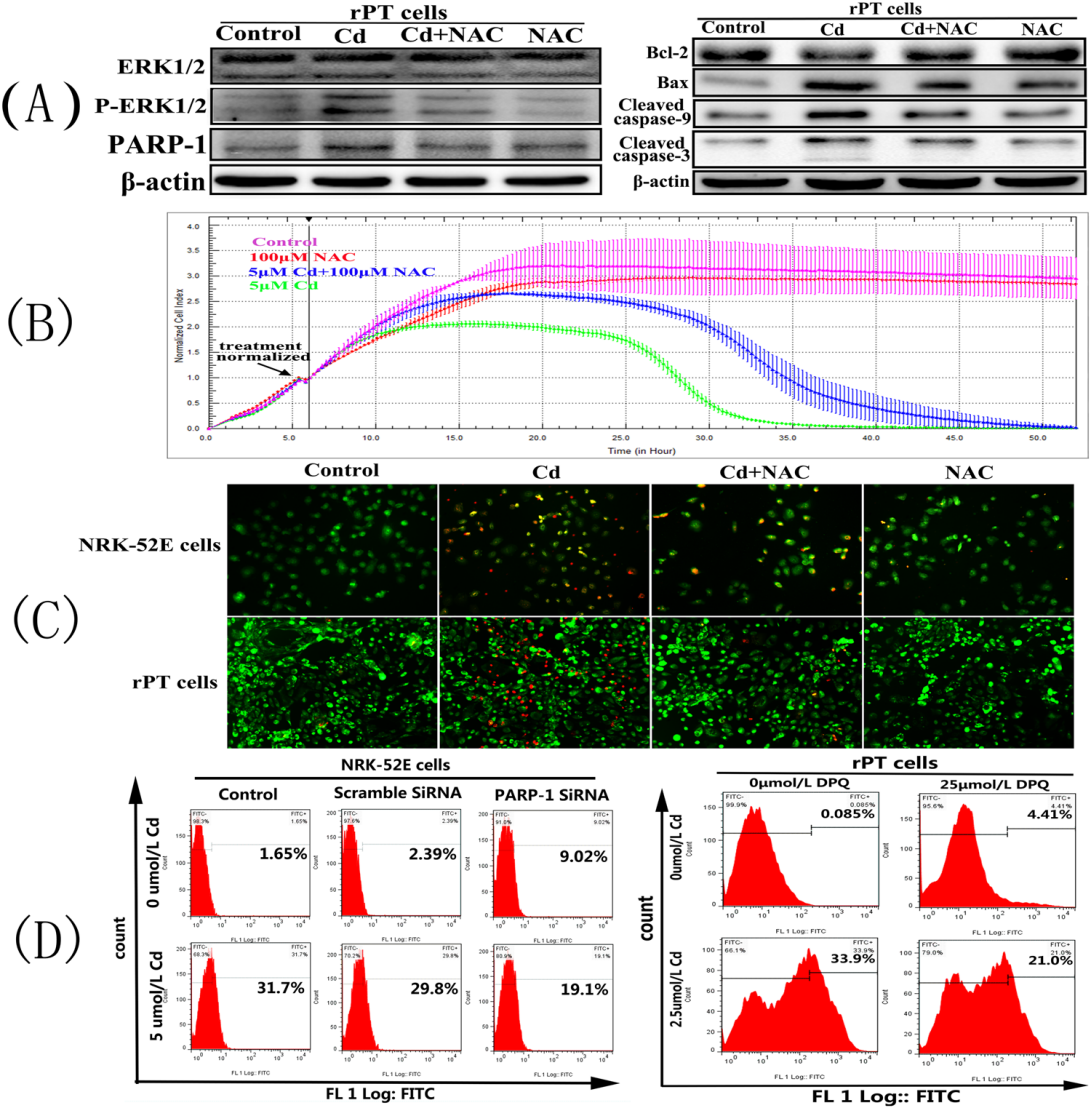
**C**d-induced oxidative stress and the effect of parthanatos on reactive oxygen species (ROS) in cells. (**A**) NAC significantly reduced the expression of related apoptotic proteins in primary rPT cells. (**B**) NAC attenuates the effect of Cd on NRK-52E cells proliferation. (**C**) AO-EB staining showed that NAC significantly reduced the Cd-induced cell apoptosis and death (200×). (**D**) Inhibition of PARP-1 overexpression or silencing its gene reduced the ROS content obviously.

Our previous study showed that Cd could cause oxidative stress^19, 20^; therefore, to verify the role of oxidative stress in parthanatos, we used a strong antioxidant, NAC, to interfere with the effect of Cd on cells. We found that compared with the Cd alone group, in cells co-treated with NAC and Cd, the levels of Bcl-2 increased significantly, and the levels of P-ERK1/2, cleaved caspase-9 /3, PARP-1, and Bax proteins were reduced significantly (Fig. 9A). Furthermore, the RTCA method was used to detect the cell proliferation in real time (Fig. 9B). The results showed that compared with the Cd group, the cells survival time in Cd+NAC group was longer. In addition, we examined the protective effect of NAC on Cd-induced apoptosis using AO-EB staining. As shown in Fig. 9C, NAC could reduce the extent of apoptosis or cell death effectively in both NRK-52E and primary rPT cells. Finally, we examined the effect of Cd and PARP-1 protein on cellular ROS levels by flow cytometry (Fig. 9D). The ROS levels in NRK-52E cells increased from 1.65 to 31.7% and the ROS levels in primary rPT cells increased from 0.085 to 33.9% after Cd treatment; however, when PARP-1 protein overexpression was inhibited, the ROS levels in Cd-treated NRK-52E cells reached only 19.1% and the levels ROS of Cd-treated primary rPT cells reached only 21%. These results indicated that Cd treatment causes oxidative stress in NRK-52E and primary rPT cells, and that inhibition of PARP-1 overexpression could partially ameliorate this Cd-induced oxidative stress.

### Activation of JNK1/2 and p38 proteins by Cd-induced parthanatos in primary rPT cells

**Figure 10:**
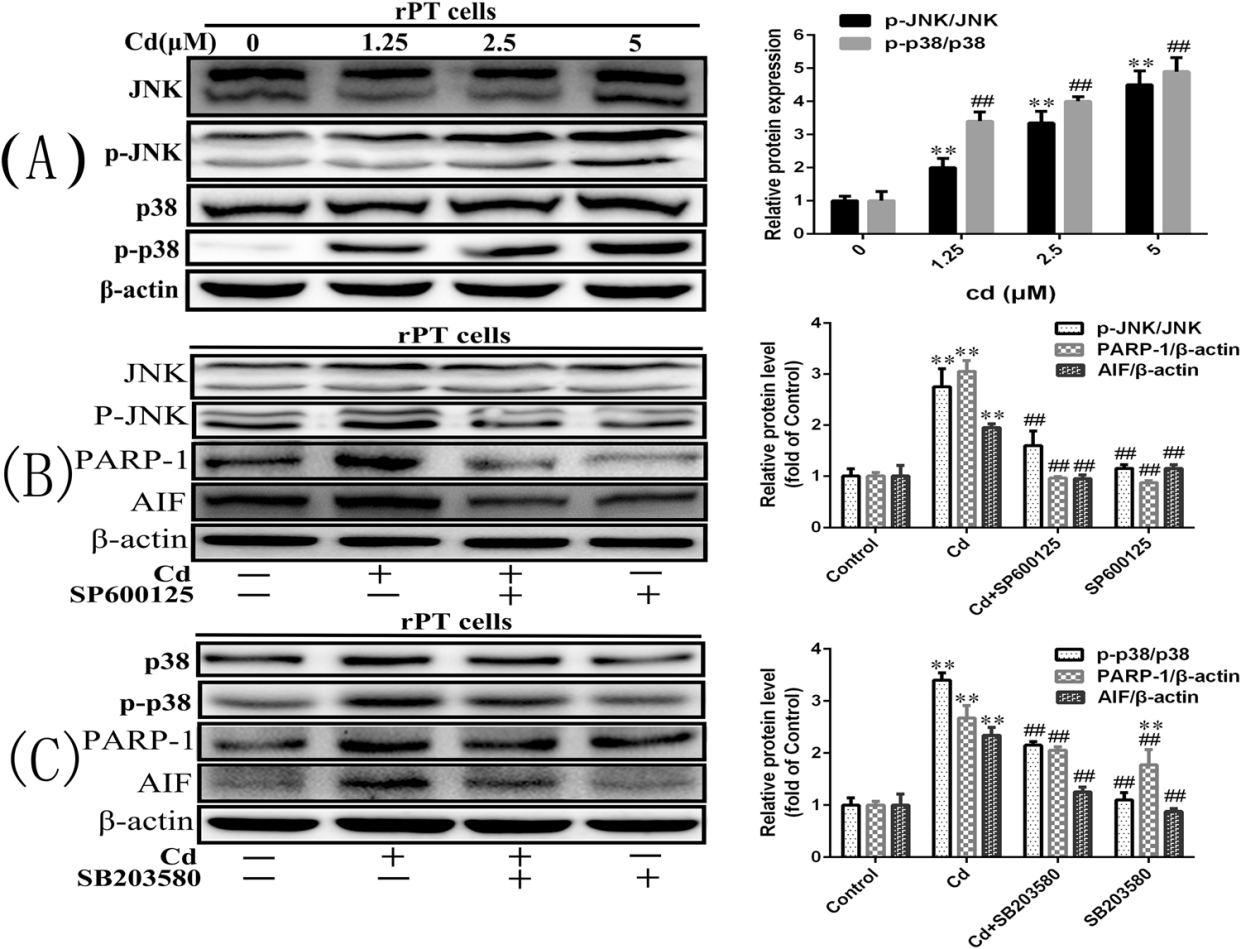
**A**ctivation of JNK1/2 and p38 protein by Cd-induced parthanatos in primary rPT cells. (**A**) Cd activates the JNK1/2 MAPK and p38 MAPK pathway in primary rPT cells. (**B** and **C**) Inhibition of p-JNK or p-p38 protein activity can reduce the expression of PARP-1 and AIF. The quantitative analysis was performed on the western blotting results of three independent experiments (mean ± SD, n = 3). Respectively, **P < 0.01 vs. the control group by one-way ANOVA. ^##^P < 0.01 vs. the Cd group by one-way ANOVA.

The results in Fig. 9 showed that Cd causes oxidative stress in rat renal tubular epithelial cells, and JNK 1/2 and p38 are reported to be activated by ROS^21, 22^. Therefore, we detected the levels of JNK1/2 and p38 proteins to explore whether the JNK1/2 and p38 MAPK pathways are involved in Cd-induced apoptosis and the pathways associated with parthanatos. The results showed that Cd upregulated the levels of p-JNK and p-P38 proteins in a concentration-dependent manner (Fig. 10A). In addition, SP600125 an inhibitor of p-JNK, and SB203580 an inhibitor of p-p38, inhibited significantly the increase in p-JNK and p-P38 protein levels induced by Cd, and reduced the levels of PARP-1 and AIF proteins significantly (Fig. 10B and C). These results indicated that Cd treatment could activate the JNK1/2 and p38 MAPK signalling pathways in primary rPT cells and that the MAPK pathway affects parthanatos by regulating the expression of the PARP-1 protein.

## Discussion

Cadmium (Cd) is a toxic heavy metal found widely in nature, and diseases caused by Cd poisoning result in serious economic losses worldwide^23, 24^. The toxicity mechanism of Cd has been reported in many kinds of cells and animal experiments^25, 26^; however, the mechanism of Cd damage to the kidneys is unclear. In this study, we used CCK-8, RTCA, and western blotting methods to confirm that Cd has strong toxic effects on renal tubular epithelial cells (Fig. 1), and then studied the mechanism of Cd’s renal toxicity.

The recently discovered PARP-1-dependent cell death pathway was named as parthanatos to distinguish it from other forms of cell death^27^. The definition of parthanatos involves two main criteria. One is that a large amount of PAR is synthesised in the process of death, and the other is that inhibition of PARP-1 overexpression or *PARP-1* gene silencing could inhibit cell death completely or partially^28^. Overexpression of PARP-1 is the most critical step to initiate parthanatos, during which PARP-1 synthesises PAR using NAD^+^ as substrate in the presence of ATP^29^. Parthanatos is reported that it is related to a variety of diseases, such as diabetes, inflammation, Brain trauma and so on^30, 31^. An important feature is the dependence on the PARP-1 protein, and previous study found that Cd treatment of renal tubular epithelial cells could increase the expression of PARP proteins significantly^32^. In addition, it was reported that PARP-1 protein activity accounts for almost 90% of the activity of the PARP protein family^33^. Therefore, we speculated that parthanatos is involved in this process. In the present study, we showed that parthanatos participates in the early phase of Cd-induced renal tubular epithelial cell injury and is associated with oxidative stress and the MAPK pathway.

Mitochondria are the main sites at which eukaryotic cells undergo oxidation and are also the main site of cellular ROS production^34^. ROS have also been implicated in PARP-1 overexpression; however, a significant increase in intracellular ROS is an important feature of oxidative stress. In addition, ROS might be involved in the expression of the target gene as a second messenger by activating the key transcription factors^35^. It was reported that Cd-induced cell damage is associated with a significant increase in ROS, and that oxidative stress can promote apoptosis and play an important role in cell damage, by damaging lipids, proteins, and DNA^36^. Apoptosis can be divided into three pathways: the death receptors-mediated pathway, the endoplasmic reticulum pathway, and the mitochondria-mediated apoptosis pathway. Among them, the mitochondria-mediated apoptosis pathway is the most important pathway^37, 38^. Mitochondria-mediated apoptosis mainly involves the release of cytochrome C and AIF, and the activation of caspase-9/3 and Bax^39^. Overexpression of PARP-1 consumes a lot of NAD^+^ and ATP, which would decrease the cellular ATP content significantly. Intracellular ATP depletion would induce changes in the cellular structure, biochemistry, and function, leading to cell death^40^. In addition, oxidative stress inhibits the synthesis of ATP and adenine nucleotide translocation^41^.

In this study, we used different concentrations of Cd to treat NRK-52E cells and primary rPT cells for different times, and then detected the levels of proteins related to the parthanatos pathway and the changes of ATP and NAD^+^ contents in cells. The results showed that the levels of PARP-1, the polymer PAR, and AIF in the nucleus were increased significantly in Cd-treated cells in a time and concentration-dependent manner (Figs 2 and 3). The ATP and NAD^+^ contents also decreased with increasing Cd-treatment time and concentration (Fig. 4). DPQ is a highly effective and specific PARP-1 inhibitor that has been used widely in related studies^42, 43^. When we used a PARP-1 siRNA to knock down the *PARP-1* gene in NRK-52E cells and used DPQ to inhibit the activity of PARP-1 in primary rPT cells: the PARP-1, PAR, and AIF levels and the extent of cell death decreased significantly (Fig. 5). This suggested that PARP-1 protein mediates Cd-induced rPT cell death via parthanatos, and this process involves a change in mitochondrial function, as well as oxidative stress.

The PARP protein is a downstream molecule in the mitochondrial apoptotic pathway, PARP activity is thought to be a marker of caspase-3 protein activation and is also an iconic event for cell apoptosis^44^. Mitochondrial proteins, such as AIF and CytC, are released from mitochondria when the mitochondrial membrane potential is reduced. AIF is transported to the nucleus where it causes DNA fragmentation^33^. In addition, CytC mediates the traditional mitochondrial apoptotic pathway. To further explore the changes in mitochondria during this process, we examined the activation of the mitochondrial apoptotic pathway (Fig. 6). The results showed that mitochondria-mediated apoptosis was activated by Cd-treated primary rPT cells. When the cells were co-treated with DPQ and Cd for 12 h, we found that the mitochondrial membrane potential was restored, normalized cell index cell was increased and mitochondria-mediated apoptosis was inhibited significantly compared to the Cd alone-treated group (Fig. 7). This suggested that PARP-1 protein acts directly or indirectly on mitochondria to alter the membrane potential and initiate apoptosis during parthanatos, and both caspase-dependent and caspase-independent apoptosis are involved in this process. It has been reported that caspase is activated in parthanatos; however, broad-spectrum caspase inhibitors did not inhibit parthanatos^45^. Therefore, it was speculated that in this process, caspase does not play a substantial role. Overexpression of PARP-1 protein mediates parthanatos, and the PARP protein, as a caspase-3/7 reaction substrate, is involved in the mitochondrial apoptosis pathway. We hypothesized that PARP-1 acts as a bridge between parthanatos and apoptosis, because PARP-1 acts on mitochondria through PAR, causing membrane potential loss and the release AIF and CytC, while mediating both caspase-dependent and caspase-independent apoptosis.

In addition, we showed that Cd treatment induces oxidative stress and that compared with the Cd alone group, inhibition of PARP-1 overexpression or silencing its gene reduced the ROS content significantly (Fig. 9D). This indicated that PARP-1 overexpression plays a role in oxidative stress in Cd-treated cells. Furthermore, when we treated the cells with the ROS inhibitor NAC, the Bcl-2/Bax ratio increased, and the levels of cleaved capase-9/3 and PARP-1 were reduced significantly (Fig. 9A) and normalized cell index cell was increased (Fig. 9B). AO-EB staining also showed that cell apoptosis and death were reduced significantly (Fig. 9C). These results indicated that oxidative stress plays an important role in Cd-induced rPT cell injury and has synergistic effects with parthanatos.

The mitogen-activated protein kinase (MAPK) signalling pathway is an important signal transduction system *in vivo*, and is involved in a variety of physiological and pathological processes, such as cell growth, inflammation, apoptosis, proliferation, and differentiation^46^. The MAPK pathway includes three major pathways: ERK MAPK, JNK MAPK, and p38 MAPK. At present, the mechanism of MAPK kinase regulation in cell apoptosis is not very clear, MAPK regulates the way of apoptosis is depend on the type of cell, the way of stimulation and the duration of the stimulus. Furthermore, there are reports that JNK is involved in parthanatos^13, 47^. In this study, we examined the effect of Cd on the ERK1/2 MAPK pathway and found that Cd activated this pathway, in addition, NAC treatment alleviated the Cd-induced ERK1/2 MAPK pathway (Figs 1B and 9A), this suggested that Cd affects the ERK1/2 pathway via oxidative stress. To further investigate the role of the MAPK pathway in Cd-induced renal tubular epithelial cell injury, we detected the levels of JNK1/2 MAPK and p38 MAPK pathway-associated proteins in primary rPT cells and showed that Cd also activated these two pathways (Fig. 10A). And both JNK1/2 and P38 can alter PARP-1 and AIF expression, thus affecting parthanatos (Fig. 10B and C).

In conclusion, we demonstrated that Cd treatment of rPT cells caused parthanatos and activation of the MAPK pathway, in which process oxidative stress and mitochondrial damage play key roles. In addition, parthanatos with oxidative stress has a synergistic effect on apoptosis, and JNK1/2 and p38 contribute to parthanatos.
